# Human H3N2 Influenza Viruses Isolated from 1968 To 2012 Show Varying Preference for Receptor Substructures with No Apparent Consequences for Disease or Spread

**DOI:** 10.1371/journal.pone.0066325

**Published:** 2013-06-21

**Authors:** Shelly Gulati, David F. Smith, Richard D. Cummings, Robert B. Couch, Sara B. Griesemer, Kirsten St. George, Robert G. Webster, Gillian M. Air

**Affiliations:** 1 Department of Biochemistry & Molecular Biology, University of Oklahoma Health Sciences Center, Oklahoma City, Oklahoma, United States of America; 2 Department of Biochemistry, Emory University School of Medicine, Atlanta, Georgia, United States of America; 3 Department of Molecular Virology and Microbiology, Baylor College of Medicine, Houston, Texas, United States of America; 4 New York State Department of Health, Wadsworth Center, Albany, New York, United States of America; 5 St Jude Children’s Research Hospital, Memphis, Tennessee, United States of America; Johns Hopkins University - Bloomberg School of Public Health, United States of America

## Abstract

It is generally accepted that human influenza viruses bind glycans containing sialic acid linked α2–6 to the next sugar, that avian influenza viruses bind glycans containing the α2–3 linkage, and that mutations that change the binding specificity might change the host tropism. We noted that human H3N2 viruses showed dramatic differences in their binding specificity, and so we embarked on a study of representative human H3N2 influenza viruses, isolated from 1968 to 2012, that had been isolated and minimally passaged only in mammalian cells, never in eggs. The 45 viruses were grown in MDCK cells, purified, fluorescently labeled and screened on the Consortium for Functional Glycomics Glycan Array. Viruses isolated in the same season have similar binding specificity profiles but the profiles show marked year-to-year variation. None of the 610 glycans on the array (166 sialylated glycans) bound to all viruses; the closest was Neu5Acα2–6(Galβ1–4GlcNAc)_3_ in either a linear or biantennary form, that bound 42 of the 45 viruses. The earliest human H3N2 viruses preferentially bound short, branched sialylated glycans while recent viruses bind better to long polylactosamine chains terminating in sialic acid. Viruses isolated in 1996, 2006, 2010 and 2012 bind glycans with α2–3 linked sialic acid; for 2006, 2010 and 2012 viruses this binding was inhibited by oseltamivir, indicating binding of α2–3 sialylated glycans by neuraminidase. More significantly, oseltamivir inhibited virus entry of 2010 and 2012 viruses into MDCK cells. All of these viruses were representative of epidemic strains that spread around the world, so all could infect and transmit between humans with high efficiency. We conclude that the year-to-year variation in receptor binding specificity is a consequence of amino acid sequence changes driven by antigenic drift, and that viruses with quite different binding specificity and avidity are equally fit to infect and transmit in the human population.

## Introduction

In a seminal series of experiments in the 1980s, Paulson and colleagues showed that the hemagglutinins (HA) of human influenza viruses show binding preference for α2–6 linked sialic acid while avian viruses bound the α2–3 linked form, and that a single amino acid change was sufficient to switch the specificity from mammals to birds and vice versa [1]. Despite the presence of α2–3 sialylated glycans in the human respiratory tract, and the efficient transmission and replication of human parainfluenza viruses that bind only α2–3 sialylated structures [2], [3], almost all human influenza viruses bind α2–6 sialylated glycans. As we and others screened human H3N2 influenza viruses, or expressed HAs, on the Consortium for Functional Glycomics (CFG) Glycan Array, we noted considerable diversity in the substructures bound by different isolates [4], [5] and significant binding of α2–3 sialylglycans has been reported in some human strains [5]–[8]. Viruses from the mid-1990s lost the ability to agglutinate chicken red blood cells, indicating a change in specificity and/or avidity [9]. The red blood cells recommended for hemagglutination-inhibition tests were changed from chicken to turkey, but by the 2000s it was widely recognized that the avidity for turkey red cells was reduced. Some of the sequence and structural changes responsible have been identified [10], but the low avidity of recent viruses has caused considerable problems with inconsistent hemagglutination-inhibition tests when seeking evidence of antigenic drift and determining when a change in the vaccine virus is needed.

Several of the published studies used egg-adapted vaccine strains or HA genes from viruses of unknown (or unreported) passage history, and this led us to a systematic investigation of the binding properties of all major variants of human H3N2 viruses from their appearance in 1968 to 2012. We sought viruses that were isolated in mammalian cells, never grown in eggs, and minimally passaged. We screened the binding of Alexa488-labeled virions on the CFG Glycan Array. We found an overall gradation in binding preference from short, branched α2–6 sialylated (NeuAc) structures to long linear or long branched polylactosamine-containing sialylated glycans. Occasionally strains appeared with very different binding patterns, including viruses with affinity for NeuAcα2–3, but these only lasted a year or two. However, recent isolates bound to NeuAcα2–3 as well as NeuAcα2–6 but the NeuAcα2–3 binding was almost entirely eliminated in the presence of oseltamivir, indicating the HA was binding NeuAcα2–6 but the NA bound NeuAcα2–3. Other recent H3N2 viruses with a mutation of D151 in the NA have been shown to bind to NeuAcα2–3 [11], [12]. Entry of these dual-specificity viruses into MDCK cells was reduced when oseltamivir was present during initial adsorption then removed to allow cell-to-cell spread, indicating that the binding by the NA can contribute to entry.

## Results and Discussion

### Properties of the H3N2 Viruses Isolated from 1968 to 2012

The 45 viruses analyzed in this study are shown in Table 1. We obtained human viruses that were isolated from 1968 to 2012 in mammalian cells and never passaged in eggs. We grew the viruses in MDCK cells (8–10 T175 flasks) and purified them by sucrose density gradient centrifugation. The HA1 coding sequences were determined for all viruses to ensure that we knew what was run on the Glycan Array. New sequences were deposited into Genbank via the NIAID Influenza Research Database (IRD) http://www.fludb.org [13]. Sequences of HA of A/Albany/11/68, A/Albany/1/69. A/Albany/1/70, A/Albany/42/75 from early passages in rhesus monkey kidney (RMK) cells were already in the database; the sequences after 2–3 passages in MDCK cells were identical, and we similarly have seen no change in Oklahoma isolates between isolation in RMK cells and after multiple passages in MDCK cells. Earlier studies also showed no change in HA sequence between patient material and MDCK cell passages [14], [15]. We always passage viruses at limiting dilution and this might be why we saw only one (R220G in one virus) of the several changes attributed to MDCK cell passage by others [16]–[18]. The relationship of the HA1 sequences to each other and to the vaccine strains (Table 1) was shown by the IRD phylogeny tool PhyML [19]. The HA1 sequences of all the viruses used are aligned in Figure S1. We determined the HA titers of viruses with human, guinea pig, chicken and turkey red blood cells, and, for recent viruses and some earlier viruses as controls, we determined if oseltamivir reduced the HA titer. This information is included in Table 1.

**Table 1:** Human H3N2 influenza isolates used in this study.

Isolate	Collectiondate	Source of virus stock	Passage History (Received)	Additional passages (for binding)	Closest vaccine sequence	Genbank Accession Number	Log_2_ HA titer with red blood cells from:				
							human	guinea pig	chicken	turkey	
							–	–	–	–	+ ost^1^
A/Albany/11/1968	1968	Wadsworth^3^	pRhMK^4^3	MDCK3	A/Aichi/2/1968	CY019891	7	8	7	8	nt^2^
A/BCM/2/1968 (HK/813558)	2/8/68	Baylor^5^	HEK^6^2, Vol^7^1, HEK1, MDCK	MDCK2	A/Aichi/2/1968	CY111495	nt	nt	nt	7	7
A/Albany/1/1969	1969	Wadsworth	pRhMK4	MDCK3	A/Aichi/2/1968	CY019899	7	4	4	6	nt
A/BCM/2/1969	1/7/69	Baylor	HEK1, MDCK1	MDCK2	A/Aichi/2/1968	CY111496	6	7	6	5	nt
A/Albany/1/1970	1970	Wadsworth	pRhMK3	MDCK3	A/Aichi/2/1968	CY022938	8	7	6	7	nt
A/BCM/1/1970	1/14/70	Baylor	HEK2, MDCK1	MDCK2	A/Aichi/2/1968	CY111497	7	0	5	7	nt
A/BCM/1/1972 (Udorn/307/72)	9/xx/72	Baylor	BEK^8^4, Vol1, MDCK1	MDCK2	A/Udorn/307/1972	CY111498	0	1	0	2	nt
A/BCM/1/1973	1/15/73	Baylor	MDCK1	MDCK2	A/Udorn/307/1972	CY111499	1	5	0	2	nt
A/BCM/1/1974 (A/Georgia/101/74)	9/9/74	Baylor	HEK2, Vol1, MDCK1	MDCK2	A/Port Chalmers/1/1973	CY111500	10	0	7	7	nt
A/Albany/42/1975	1975	Wadsworth	pRhMK2	MDCK4	A/Victoria/3/1975	CY021077	7	7	6	7	nt
A/BCM/3/1975	1/11/75	Baylor	MDCK3	MDCK2	A/Victoria/3/1975	CY111501	6	7	5	7	nt
A/BCM/1/1976	1/xx/76	Baylor	MDCK3	MDCK2	A/Victoria/3/1975	CY111502	5	5	1	4	nt
A/BCM/11/1976	1/28/76	Baylor	MDCK1	MDCK2	A/Texas/1/1977	CY111510	9	10	8	9	nt
A/BCM/3/1977	12/30/77	Baylor	MDCK3	MDCK2	A/Texas/1/1977	CY111503	8	8	7	7	nt
A/BCM/1/1978	1/3/78	Baylor	MDCK3	MDCK2	A/Bangkok/1/1979	CY111504	7	7	7	6	nt
A/BCM/1/1980	2/26/80	Baylor	MDCK2	MDCK2	A/Bangkok/1/1979	CY111505	8	10	8	8	nt
A/BCM/1/1981	1/5/81	Baylor	MDCK2	MDCK2	A/Bangkok/1/1979	CY111506	6	5	2	6	nt
A/BCM/1/1982	11/22/82	Baylor	MDCK2	MDCK2	A/Bangkok/1/1979	CY111507	8	7	9	9	nt
A/Memphis/33/83	1983	St. Jude^9^	MDCK1	MDCK2	A/Philippines/2/82	CY009052	7	6	7	7	nt
A/Memphis/2/1985	1985	St. Jude	MDCK1	MDCK2	A/Mississippi/1/85	CY009068	7	8	7	6	nt
A/Memphis/2/1986	1986	St. Jude	MDCK2	MDCK3	A/Leningrad/360/86	CY011144	8	6	3	5	nt
A/Memphis/3/1988	1988	St. Jude	MDCK2	MDCK2	A/Shanghai/11/87	CY111508	8	9	7	7	nt
A/Memphis/7/1990	1990	St. Jude	MDCK2	MDCK2	A/Shanghai/16/89	CY008740	3	6	5	5	nt
A/BCM/1/1991	11/25/91	Baylor	LLC^10^1, MDCK1	MDCK2	A/Beijing/353/89	CY111509	5	6	2	4	nt
A/BCM/2/1992	1/2/1992	Baylor	X^11^MDCK1	MDCK2	A/Beijing/353/89	KC539109	nt	nt	nt	7	7
A/BCM/1/1993	12/20/93	Baylor	MDCK1	MDCK2	A/Jo’burg/33/94	CY111511	6	0	5	7	nt
A/Memphis/7/1994	1994	St. Jude	MDCK1	MDCK2	A/Jo’burg/33/94	CY111512	2	1	0	0	nt
A/New York/696/1994	12/30/94	Wadsworth	pRhMK2	MDCK2	A/Jo’burg/33/94	CY011336	nt	nt	nt	5	nt
A/Memphis/9/1995	1995	St. Jude	MDCK1	MDCK2	A/Jo’burg/33/94	CY111513	5	0	3	4	nt
A/Memphis/9/1996	1996	St. Jude	MDCK1	MDCK2	A/Wuhan/359/95	CY111514	6	0	2	3	nt
A/Oklahoma/5098/1996	Late 1996	OUMC^12^	pRhMK2	MDCK>3	A/Wuhan/359/95	CY073706	2	0	3	2	nt
A/Oklahoma/3003/1996	Late 1996	OUMC	pRhMK2	MDCK5	A/Sydney/5/97	CY073705	5	0	6	6	nt
A/Memphis/5/1997	1997	St. Jude	MDCK1	MDCK3	A/Sydney/5/97	CY111515	8	7	0	6	nt
A/Memphis/14/1998	1998	St. Jude	MDCK1	MDCK3	A/Sydney/5/97	CY111516	5	2	0	0	nt
A/Memphis/49/1999	1999	St. Jude	MDCK1	MDCK3	A/Panama/2007/99	CY111517	6	0	2	7	nt
A/BCM/1/2001	11/30/01	Baylor	MDCK3	MDCK2	A/Fujian/411/02	CY111518	7	6	0	7	7
A/BCM/1/2002	1/14/02	Baylor	pRhMK2, MDCK1	MDCK1	A/Fujian/411/02	CY111519	7	0	0	8	8
A/Memphis/27/2003	2003	St. Jude	MDCK1	MDCK2	A/Fujian/411/02	CY111520	6	0	1	6	2
A/Oklahoma/323/2003	10/x/03	OUMC	pRhMK	MDCK3	A/Fujian/411/02	DQ059385	5	0	0	6	7
A/Oklahoma/1992/2005	3/21/05	OUMC	pRhMK2	MDCK3	A/California/7/2004	FJ975056	6	0	3	6	nt
A/Oklahoma/309/2006	1/12/06	OUMC	pRhMK2	MDCK4	A/Wisconsin/57/05	CY111521	7	7	5	7	6
A/Oklahoma/483/2008	1/20/08	OUMC	pRhMK	MDCK5	A/Brisbane/10/07	FJ975059	9	7	7	7	4
A/Oklahoma/5342/2010	11/20/10	OUMC	pRhMK	MDCK3	A/Perth/16/09	CY080268	6	1	1	5	1
A/Oklahoma/5386/2010	11/22/10	OUMC	pRhMK	MDCK3	A/Perth/16/09	CY0808269	6	6	4	5	2
A/Oklahoma/2280/2012	03/07/12	OUMC	pRhMK	MDCK2	A/Victoria/361/2011	KC491731	nt	nt	nt	7	2

1 Ost = oseltamivir carboxylate.

2 nt: not tested.

3 Wadsworth Center- Griffin Laboratories, New York State Department of Health, Albany, New York.

4 Primary Rhesus monkey kidney cells.

5 Baylor College of Medicine, Houston, Texas.

6 Human embryonic kidney cells.

7 Human volunteer passage.

8 Bovine embryonic kidney cells.

9 St. Jude Children’s Research Hospital, Memphis, Tennessee.

10 LLC-MK2 continuous cell line.

11 Previous tissue culture passages unknown.

12 Oklahoma University Medical Center, Virology Laboratory, Children’s Hospital.

### Binding to the CFG Glycan Array Correlates with Red Blood Cell Avidity

The purified viruses were labeled with Alexa488 and run at three concentrations on the Consortium for Functional Glycomics Glycan array, versions 5.0 (611 glycans) or 5.1 (610 glycans). The only difference between the two array versions is omission of one glycan from v5.1 that was represented twice on v5.0 with different linkers. Here we present the results using v5.1 identification numbers. The complete list of glycans on the array is given at www.functionalglycomics.org. Individual binding sites on HA have low (mM) affinity for glycans [20] and so the signals from both glycan array binding [21] and red blood cell binding measure avidity of multiple HA molecules to multiple ligands. We diluted the Alexa-labeled viruses well below the saturation level for the array so by running three concentrations we could distinguish high avidity from low avidity binding. We saw very little non-specific binding in these experiments using well-purified virus and a careful titration of Alexa488.

**Figure 1:**
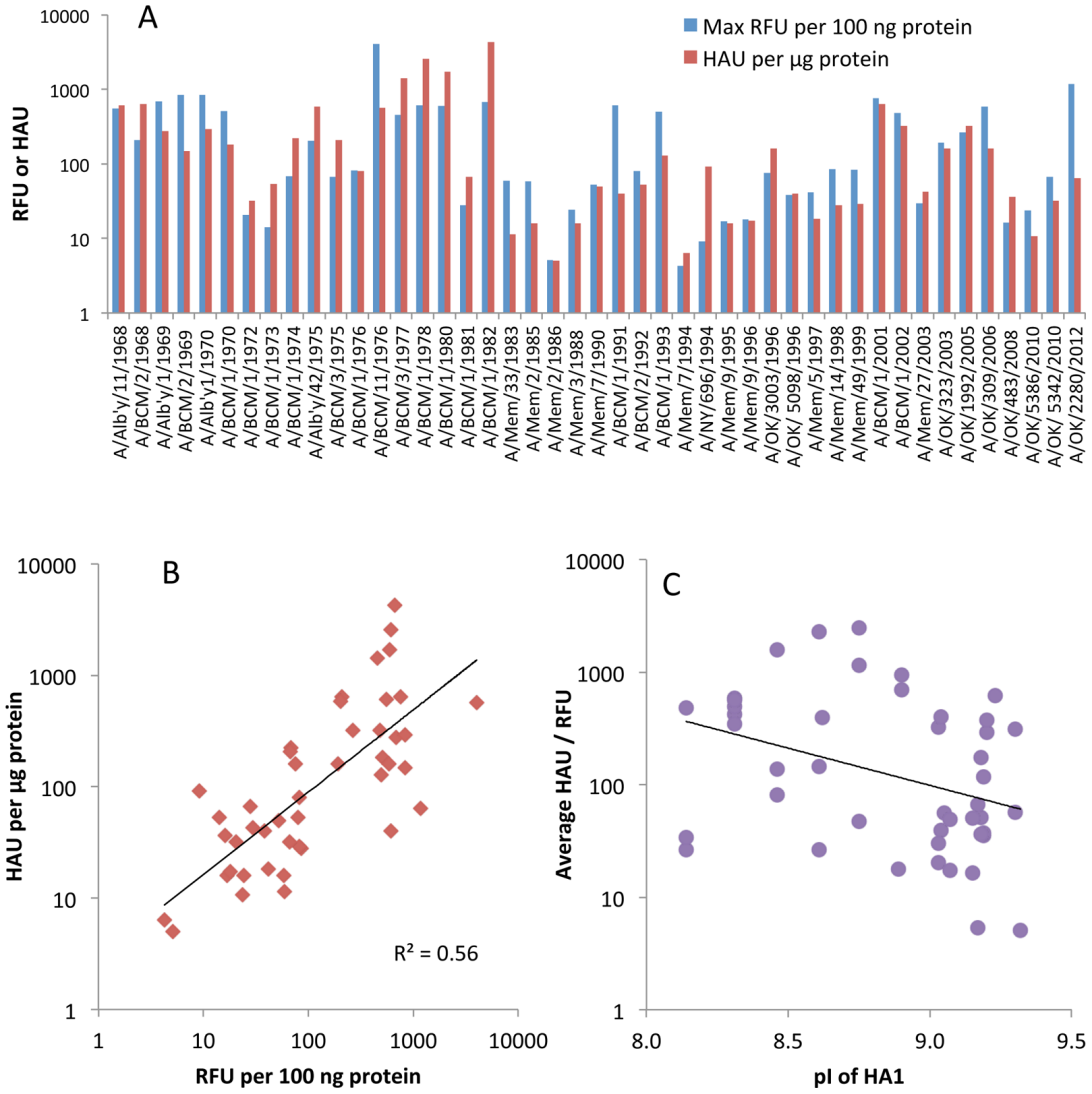
Relationship between agglutination of turkey red blood cells and the highest binding signal on the Glycan Array. A. Hemagglutinating units (HAU, red) or fluorescent signal (RFU, blue) are shown per µg or per 100 ng viral protein, respectively, to approximately equalize the magnitude. B. HAU plotted against RFU with the trendline shown in black. Note that both axes are on a log scale. C. Plot of isoelectric point (pI) against binding avidity (the average of HAU and RFU).

The 610 glycans on the array are only a small fraction of the possible receptors in the human respiratory tract. As a test of relevance of the glycan array results, we compared the signals from the array with HA titers of the 45 viruses. It is well known that influenza viruses vary in their binding to the red blood cells used as surrogate receptors in vaccine efficacy studies, so we first determined the HA titer of the purified virus preparations on human, guinea pig, chicken and turkey red blood cells (Table 1). Different patterns were seen but all viruses agglutinated turkey red blood cells, so we used the turkey red cell HAU per µg viral protein to compare with the fluorescent signal (RFU) of the highest binding glycan (Figure 1). To bring the results to a similar scale the RFU was calculated per 100 ng viral protein. Figure 1 shows two properties of the viruses; the relative avidities of binding among the 45 viruses, and the binding to red blood cells compared to glycans on the array for each individual virus. It is clear that some viruses bind strongly to both red blood cells and glycans while other show weak binding in both assays, despite the relatively high errors due to the two-fold dilutions in the HA titration and uncertainty in the viral protein applied to the array due to incorporated host cell proteins [22] or incomplete purification. Differences in avidity to red blood cells could be due to differences in HA density [5], but previous studies with viruses and purified HAs have shown clear differences in avidity between different HAs [7], [17], [18]. Figure 1 is plotted on a log scale, and shows there is up to 1000-fold variation in avidity of binding either to array glycans or to red blood cells among these H3N2 viruses. While there is an overall loss in avidity over the years, there is also a cyclical pattern of higher and lower avidities. The HAU measures viral particles while RFU measures amount of HA bound, but for most viruses there is a correlation between glycan array signal and hemagglutination (Figure 1B) suggesting that the difference in how glycans are displayed on the array compared to the red cell surface is not affecting the global results.

One obvious concern was whether the Alexa labeling would interfere with binding. Alexa succinimidyl ester reacts with epsilon amino groups of lysine residues and could cause steric blocking or interfere with ionic interactions. In addition, the number of basic amino acids in H3 HA has been noted to increase over time [23], [24] and reaction of lysine groups with Alexa488 might decrease binding to sialic acid. We used the EMBOSS program Pepstats [25] to determine the isoelectric point of the 45 HA1 sequences and confirmed the increase in basicity over time (Figure S2) but this has not increased the avidity of binding to sialylated glycans or red blood cells (Figure 1C). Two lines of evidence indicate that the Alexa labeling does not interfere with binding. First, we determined by mass spectrometry that only a subset of lysine residues were labeled in one H3N2 and two H1N1 isolates, and none of the labeled lysines are near the binding site [26]. This result is very similar to previous work, in which high concentrations of reagents that modify lysine, tyrosine or histidine residues had no effect on HA titer or antigenicity of a 1971 H3 HA [27]. Second, we compared our glycan array results for Alexa-labeled 2009 H1N1pdm virus with several published glycan binding experiments of the unlabeled low passage virus, or its expressed HA, and found no significant differences [26]. The CFG database (http://www.functionalglycomics.org) contains several glycan array results for unlabeled H3N2 viruses or expressed H3 HAs, but they all appear to be the egg-adapted, high growth vaccine version rather than cell-passaged isolates, so differences would be expected.

### Binding Specificities from 1968 to 2012

**Figure 2:**
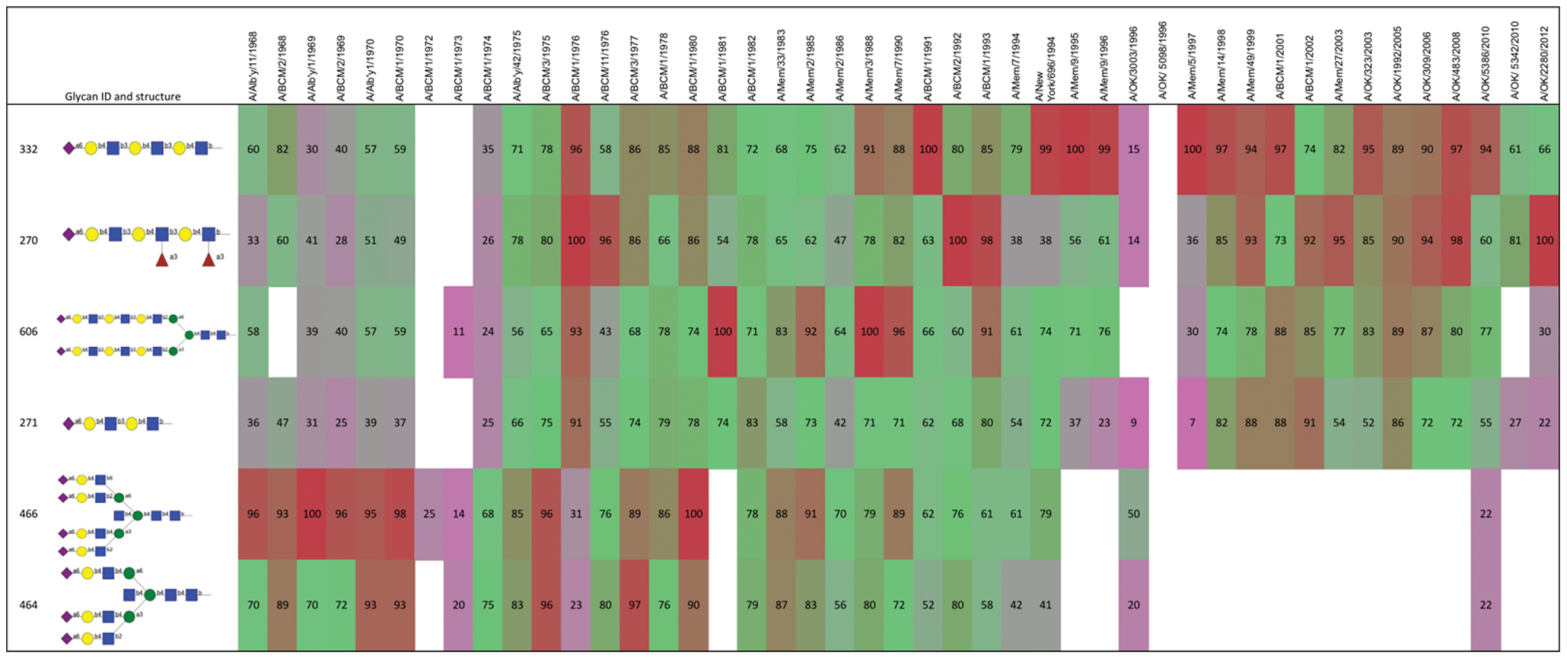
Binding profile of the top six glycans (ranked by the sum of the percentile signals, top to bottom) to viruses from 1968 to 2012 (left to right). The percentile binding of each glycan to each virus is shown, color-coded from 100 (red) to 10 (violet). White cells indicate binding less than 10% of the maximum. The colors show a shift from short branched sialylated structures in early viruses to long linear or long branched glycans in later isolates.

The binding profiles on the array were analyzed by the GlycoPattern GBP Cross Analysis program (https://glycopattern.emory.edu), which ranks binding of each glycan from high to low for the three virus concentrations, averages the rank and computes the percentile rank. To compare binding of individual glycans we summed the percentile rank of each glycan for all 45 viruses and sorted the glycans from highest to lowest summed score. The top six scoring glycans are shown in Figure 2. Binding is color coded from red (100^th^ percentile) to violet (10^th^ percentile) with binding percentile less than 10 considered insignificant. The complete results are shown in Table S1 except we deleted as insignificant the glycans that fell below an aggregate score of 15 (the top score is 3255).

First, there is no glycan out of the 610 that bound to all H3N2 viruses. There are 171 sialylated glycans on the array including 54 containing NeuAcα2–6 and 83 containing NeuAcα2–3. The top scoring four glycans (Figure 2) bound 40–42 of the 45 viruses to some extent, although these are not the preferred ligands for the early isolates. There are 30 glycans that bind detectably to 50% or more of the 45 viruses but the overall binding score of the 30^th^ glycan has dropped to 25% of the top binding glycan score. There are several NeuAcα2–6 glycans that bind very poorly and three did not bind enough to any virus to be included in Table S1 (#135 NeuAcα2–6(Galβ1–3)GalNAcα-Sp8, #480 NeuAcα2–6Galβ1–4GlcNAcβ1–6GalNAcα-Sp14, and #520 NeuAcα2–6Galβ1–4GlcNAcβ1–2Man-Sp0). Viruses isolated in 1972, 1973 and 1986 bound to several non-sialylated glycans (Table S1), but the overall binding was very low (Figure 1A) and the significance of these is uncertain.

The binding patterns in Table S1 can be divided into six phases of different specificity of binding. We discuss these from earliest to latest isolates.

**The first phase, 1968–1970:** The HA1 sequences show two sites of variable glycosylation, at Asn63 and Asn81, but are identical after amino acid 81 of HA1. The binding patterns are not significantly different. The highest binding glycans are short, branched NeuAcα2–6 structures, with lower binding to longer branched or long linear chains.

**The second phase 1972–74:** The 1972 and 1973 viruses show very little binding except to glycan #138 which is the branched pentasaccharide milk sugar LSTb, a structure that is not known to be present on cell surfaces although it has been detected as a glycosphingolipid in human meconium [28]. There are 9 amino acid changes in HA1 compared to the 1968–70 viruses. BCM/1/1972 was isolated from a human volunteer infected with A/Udorn/307/72 and the HA1 sequence is identical to Udorn/307/72. The avidity of BCM/1/1972 is very low and we thought the result might be due to the unusual passage of Udorn/307/72 in bovine cells (Table 1), but BCM/1/1973 was an original isolate passaged only in MDCK cells that had only one amino acid difference in HA1 from Udorn/72, and the same low avidity and binding pattern. The 1974 isolate, from a human volunteer passage, shows somewhat higher and more diverse binding, and has an unusual preference for two glycans that contain NeuAcα2–6GlcNAcβ1–4GlcNAc (#366 and #367). There are 7 amino acid differences in HA1 compared to the 1972/1973 strains.

**The third phase, 1975–1986:** Most of these viruses show the same preference as the earlier isolates for short sialylated branches but they now have acquired binding to longer branches and linear structures containing N-acetyllactosamine (LacNAc) repeats. They bind most of the NeuAcα2–6 glycans on the array, although with varying avidities. BCM/1/81 stands out as showing less binding to tri- and tetra-antennary glycans and its avidity to array glycans or red blood cells is lower than BCM/1/1980 (Figure 1). There are no sequence differences in HA1 between the 1981 and the 1980 isolates, so the binding differences may be due to a different distribution of HA on the virus that perhaps led to steric inhibition of binding to the highly branched glycans by the 1981 isolate. We previously noted differences in ability to bind chicken red cells depending on how the HA was displayed [5]. The overall avidity for both red cells and array glycans decreased again in 1983–1986 viruses.

**Figure 3:**
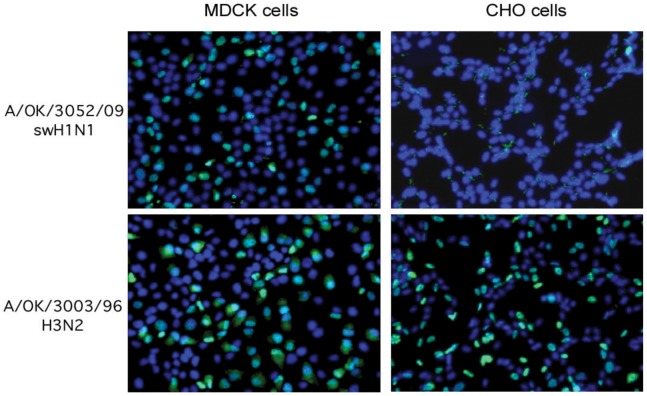
A/OK/3003/96 binds NeuAcα2–3 glycans and is able to infect CHO cells that lack NeuAcα2–6 sialylation. Virus was added to cell monolayers, then infection medium added and the infected cells incubated at 37°C for 18 hours. The cells were fixed and permeabilized for immunodetection of NP (green) accumulated in nuclei (blue DAPI) of infected cells. A control H1N1 virus that binds only NeuAcα2–6 glycans infected MDCK cells but not CHO cells.

**The fourth phase, 1988 to 1996:** These viruses acquired increased preference for long linear NeuAcα2–6(LacNAc)_n_ structures with decreasing binding to highly branched or short glycans and essentially no binding to these by 1996. Two 1996 isolates from Oklahoma do not bind either long or short branched α2–6 sialylated glycans; they bind the NeuAcα2–3 glycan sulfo-sialyl-Lewis^x^ (NeuAcα2–3Galβ1–4(Fucα1–3)(6S)GlcNAcβ-) and its un-fucosylated relative, but showed only very weak binding to other glycans. We tested the relevance of this unusual binding pattern to infection of cells. A/Oklahoma/3003/1996 was able to infect Chinese hamster ovary (CHO) cells as measured by amplification of viral nucleoprotein and its accumulation in the nucleus of infected cells (Figure 3). Since CHO cells do not express measurable α2,6-sialyltransferase (EC 2.4.99.1) [29], this suggests that OK/3003/1996 can infect cells using NeuAcα2–3 receptors.

**The fifth phase, 1997 to 2008:** These viruses bind strongly to long linear NeuAcα2–6(LacNAc)_3_ and have lost binding to short branched structures. Most of these viruses do not bind to chicken red blood cells as was reported at the time [30], [31], necessitating changes in the standard HA and HAI protocols, that now specify “either chicken, human, turkey or guinea pig” red blood cells can be used, reflecting the variability of red blood cell binding seen in Table 1. The glycan array binding pattern is in accord with a recent analysis of the N linked glycans on chicken red blood cells that showed only short branched structures and an absence of LacNAc repeats [32].

**Figure 4:**
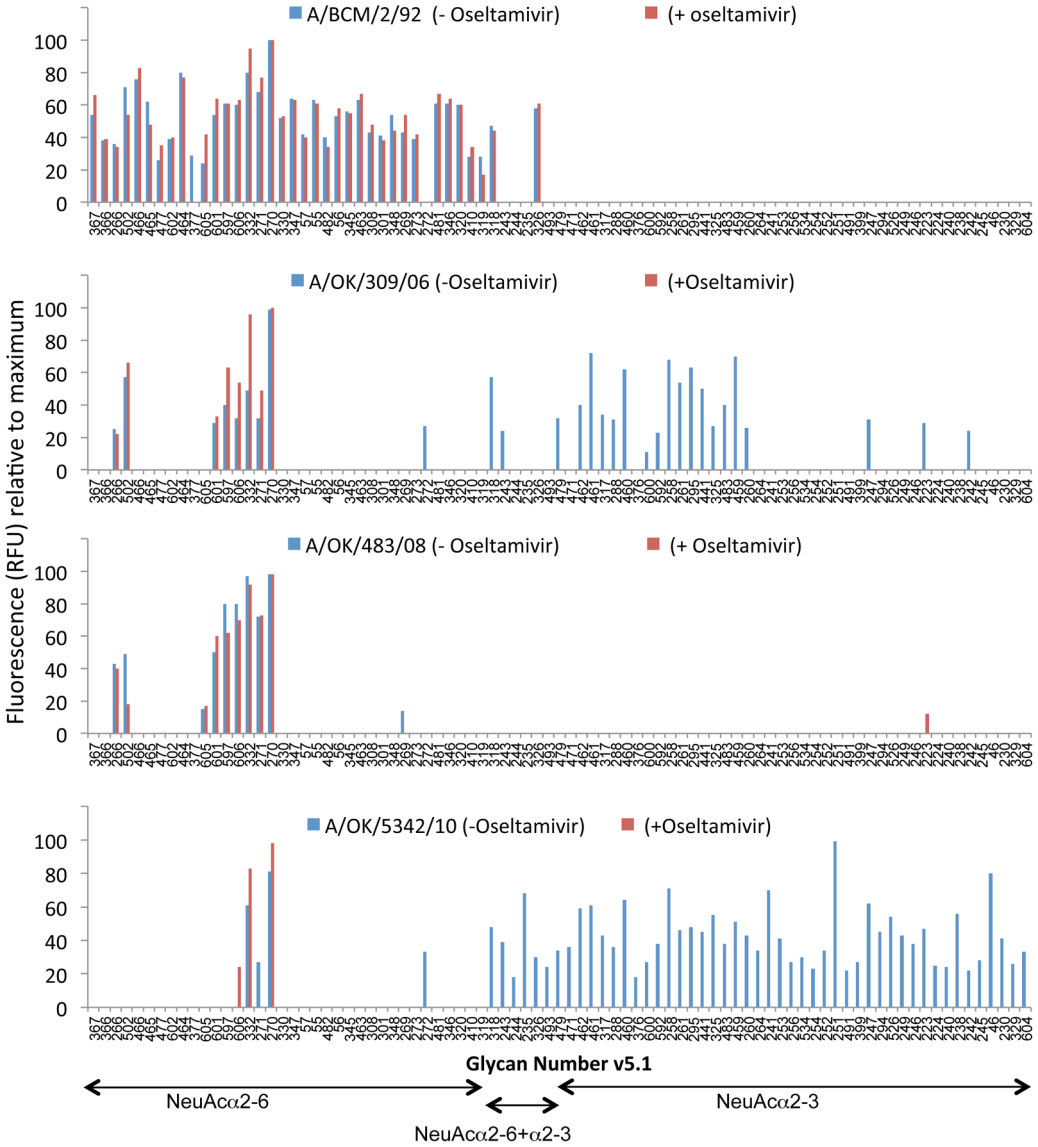
Binding of four viruses to the glycan array in the absence or presence of oseltamivir. BCM/2/92 (A) and OK/483/2008 (C) bind only NeuAcα2–6 glycans and there is no change in the presence of oseltamivir. OK/309/2006 (B) and OK/5342/2010 (D) show binding to many NeuAcα2–3 glycans but this binding is lost in the presence of oseltamivir, indicating it is NA, not HA, that binds to NeuAcα2–3.

**The sixth phase, 2010 to 2012:** We did not obtain any H3N2 isolates in 2009, but Lin et al. showed that failure of hemagglutination-inhibition (HAI) tests for 2009 H3N2 strains was due to a mutation in the NA of D151G that caused the NA to bind to substrate without cleaving it. This allowed the viruses to bind to red blood cells in an oseltamivir-inhibitable manner while anti-HA antibodies had little effect [11]. Zhu et al. followed up with glycan array and crystal structure analysis of expressed NA from A/Tanzania/205/2010 (H3N2) with either Asp or Gly at position 151. They conclusively showed that the NA with G151 binds to NeuAcα2–3 sialylated glycans but does not cleave them [12]. The isolates we obtained in 2010 and 2012 all bind a variety of NeuAcα2–3 containing structures, and in most cases show higher binding to these “avian” type receptors than to any NeuAcα2–6 ligands (Table S1). We therefore screened these and other selected viruses from our collection on the CFG Glycan Array in the presence or absence of the neuraminidase inhibitor oseltamivir carboxylate (Figure 4). Oseltamivir had no effect on binding of viruses that only bind α2–6 sialylated glycans (A/BCM/2/92 and A/Oklahoma/483/2008; also A/Memphis/27/2003 and Oklahoma/323/2003, not shown), but oseltamivir inhibited all binding to α2–3 sialylated glycans by A/Oklahoma/309/2006 and A/Oklahoma/5342/2010 (Figure 4) and also A/Oklahoma/2280/2012 (not shown), showing that the α2–3 binding is by NA and not HA. The NA of A/Oklahoma/309/2006 has a mutation of Asp to Glu at 151. We previously showed that in influenza B NA, mutation D151E (N2 numbering) reduced k_cat_ by 10-fold but at the same time reduced Km by 6-fold, showing tighter binding to substrate and little cleavage activity even with this conservative change [33]. The NA sequences of both A/Oklahoma/5342/2010 and A/Oklahoma/2280/2012 showed a mixed population of approximately 50% Asp and 50% Asn at 151.

### Does Binding by Non-cleaving NA Assist in Virus Entry into Cells?

To determine if the α2–3 sialic acid binding by the 151 variant NA can be used by the virus to infect cells, we mixed virus with oseltamivir and added it to MDCK cells for 1 hr at room temperature for the virus to adsorb, then aspirated off the virus and inhibitor and added infection medium without oseltamivir. The results are shown in Table 2. Oseltamivir inhibited entry of OK/2280/2012 and OK/5342/2010, while there was low inhibition of OK/309/2006 and no effect on BCM/3/68, a virus that binds only NeuAcα2–6 glycans. These results suggest that the NA binding to α2–3 glycans is the major route of entry for the 2012 and 2010 viruses. There is less effect on entry of OK/309/2006, which was one of the viruses that deleted most of the NA coding sequences on passage in MDCK cells resulting in resistance of multi-cycle growth to oseltamivir [4]. The partial inhibition of entry of OK/309/2006 by oseltamivir shown in Table 2 likely indicates that entry is mediated by a combination of HA and NA binding.

**Table 2:** Effect of oseltamivir on virus entry.

	A/BCM/3/68	A/OK/309/06	A/OK/5342/10	A/OK/2280/12
Virus titer (TCIU/ml)	2×10^6^	2×10^6^	2×10^4^	2×10^5^
Effective titer in the presence of 10 µM oseltamivir (TCIU/ml)	2×10^6^	2×10^5^	<10	<10
Conc. oseltamivir that inhibited virus entry (µM)	>10	1.0	0.01	0.01

Viruses were adsorbed to cells in the presence or absence of oseltamivir for 1 hr, then the inoculum aspirated off, infection medium without drug was added and the plates incubated for 3 days to allow virus growth. Both virus and oseltamivir were titrated at 10-fold dilutions so the errors are ±1 log.

### Why so much Variation in Binding?

It is widely accepted that the genetic evolution of human influenza HA is driven by selection of mutants that escape antibody neutralization while retaining ability to infect and transmit among humans. It has also been known for 30 years that the main binding sites of neutralizing antibodies surround the receptor binding site, so it is not surprising that some of the antibody escape mutations change binding properties (“adsorptive mutants” as described by Fazekas de St Groth [34]). It has been suggested that an alternative model might be that receptor binding drives antigenic drift [35], but we are not aware of data that would support this model in the natural evolution of human influenza viruses. The authors suggested that the receptor-driving model might help answer the question of how viruses can escape from polyclonal antisera, but we and others have shown there is immunodominance of particular antigenic sites recognized by human antibodies and the immunodominant site can change as the virus evolves [36]–[39]. There are a vast array of sialylated glycan structures on the surface of the human respiratory tract [40] and so changes in binding specificity or even avidity might not be expected to impact viral infectivity. That seems to be the case with the H3N2 viruses, since despite large differences in specificity and avidity of binding glycans and red blood cells over time, they all rapidly spread around the world.

We tried to correlate specific sequence changes with differences in binding, but did not identify simple relationships. Except for BCM/1980 and BCM/1981, all of the viruses showed multiple sequence differences and sophisticated docking programs might be needed to explain the differences in binding specificities.

### Summary and Conclusions

None of the 610 glycans on the array (166 sialylated glycans) bound to all viruses; the closest was NeuAcα2–6(Galβ1–4GlcNAc)_3_ in either a linear or biantennary form, that bound 42 of the 45 viruses. The earliest viruses show a preference for short, branched sialylated glycans while recent viruses bind better to long polylactosamine chains terminating in sialic acid. Viruses isolated in 1996, 2006, 2010 and 2012 bind glycans with α2–3 linked sialic acid; for 2006, 2010 and 2012 viruses the binding to the NeuAcα2–3 array glycans was inhibited by oseltamivir while binding of the α2–6 glycans was not affected by oseltamivir, indicating it is only the NA that is binding α2–3 linked sialic acid while the HA binds NeuAcα2–6 glycans. More significantly, oseltamivir inhibited entry of 2010 and 2012 viruses into MDCK cells, indicating that the virus can use NeuAcα2–3 glycans as entry receptors.

All of the viruses we studied were representative of epidemic strains that spread around the world, so all could infect, cause disease and transmit between humans with high efficiency. We conclude that the year-to-year variation in receptor binding specificity is a consequence of amino acid sequence changes that have been driven by antigenic drift, and that viruses with quite different binding specificities and avidities are equally fit to infect and transmit in the human population.

## Materials and Methods

### Viruses and Purification

The sources and passage histories of the H3N2 viruses used in this study are included in Table 1. The viruses were grown in Madin-Darby canine kidney (MDCK) cells in DMEM:Ham's F12 medium (1∶1) with ITS+ (BD Biosciences) and 0.5 µg/ml trypsin (TPCK treated, Worthington). All the virus stocks were first grown at 10-fold dilutions in six-well plates of MDCK cells, then an aliquot from the limiting dilution well was used to inoculate 6–10 175cc flasks. After incubation at 37°C for 3 days, the supernatants were clarified by low speed centrifugation. The viruses were pelleted from the supernatant then purified by centrifugation through a 10% to 40% sucrose gradient, pelleted, and resuspended in borate buffered CaMg saline (0.15 M NaCl, 0.2 5 mM CaCl_2_, 0.8 mM MgCl_2_, 20 mM boric acid, 0.13 mM Na borate, pH 7.2). Viruses were quantified by hemagglutination assay and total viral protein (Bio Rad protein assay).

### Hemagglutination Assay

The purified viruses were two-fold serially diluted in 50 µl of PBS and 50 µl of washed turkey (0.6%), chicken (0.6%), guinea pig (0.6%) or human (0.6%) red blood cells added. The plates were kept at 4°C and hemagglutination was read at 60 min. In some cases, oseltamivir carboxylate (0.02 µM) was added to the virus dilutions to inhibit agglutination caused by binding in the active site of the NA.

### Isolation of Viral RNA, Reverse Transcription, and PCR Amplification (RT-PCR)

The HA1 coding regions of all the viruses used in this study were sequenced immediately before the large-scale growth passage. For RNA extraction, the virus-containing medium was cleared of cell debris (3,000 g for 5 min) then virus was concentrated by sedimentation (SW28 rotor, 25,000 rpm for 2 hr at 4°). The virus pellet was resuspended in borate buffered CaMg saline. Viral RNA was isolated from the pelleted virus using the QiAmp Viral RNA extraction mini kit (QIAGEN, Cat. # 52904). cDNA was synthesized using the Omniscript RT kit (QIAGEN, Cat. # 205111) and an oligodeoxynucleotide (5'-AGCAAAAGCAGG) that is complementary to the 12 conserved nucleotides at the 3' end of influenza type A viral RNA segments. To amplify the HA1 gene of all H3N2 isolates a pair of H3 HA specific primers were used: 5′- AGCAAAAGCAGGGGAT and 5′-CGTACCAACCGTCTACCATTC. The PCR products were separated by electrophoresis on a 1% agarose gel and extracted using the QiA Quick Gel Extraction kit (QIAGEN, Cat. #28704). The purified RT-PCR products were sequenced at the Oklahoma Medical Research Foundation sequencing Facility using an ABI 3730 Capillary Sequencer with H3HA specific primers.

### Alexa Labeling of Viruses

The viruses were purified to an HA titer of about 1.0×10^5 ^HAU/ml. To 100 µl (∼1.0×10^4 ^HA units) of virus was added 10 µl of 1.0 M sodium bicarbonate pH 9.0. Alexa Fluor-488 succinimidyl ester (Molecular Probes Cat. # A20000) was added in a ratio of 0.005 µg Alexa per HAU, determined by HA titration to give sufficient labeling without loss of binding activity [26]. After stirring for 1 hr at room temperature in the dark, the sample was dialyzed (Slide-A-Lyzer Mini Dialysis Units 7000 MWCO, Pierce) in borate buffered CaMg saline at 4°C overnight. An aliquot of the dialyzed virus was run on a 10% SDS-PAGE gel to confirm fluorescence of HA1 with no visible labeling of internal viral components or non-viral proteins. The HA activity of labeled virus was checked again to make sure binding was not reduced by the conjugation of Alexa.

### Glycan Array Analysis and Data Processing

The 45 viruses used in this study were run at 3 different concentrations on v5.0 or v5.1 of the CFG Glycan Array using the buffers and conditions described previously [41]. The binding was done at 4°C where neuraminidase (NA) activity is undetectable but selected viruses were also screened in the presence of the neuraminidase inhibitor oseltamivir (50 nM). For each virus an initial dilution was made according to the HA titer that was predicted to give a low binding signal, applied to the glycan array slide, washed and read, then higher concentrations were applied to the same slide until there were data in the linear range for three concentrations of virus. For data analysis we used the method described by Heimburg-Molinaro et al [41] to take into account the differing avidities. At each concentration the glycans are ranked from highest to lowest, then for each glycan an average rank is computed from the three concentrations and that rank converted to a percentile. The program to do this (“GBP Cross Analysis”) is available as part of the GlycoPattern suite (http://glycopattern.emory.edu) at Emory University that also includes the motif miner GLYMMR [42]. Based on the percentile of all 45 viruses at three concentrations, we calculated the score per glycan and ranked the glycans from highest to lowest binding scores.

### Infectivity Assays

**Virus entry into CHO cells:** CHO cells were grown on cover slips in 35 mm plates. The cells were washed three times with CaMgPBS and infected with 300 µl freshly grown virus from MDCK cells. The cells were incubated at 4° for 2 hr to allow the virus to bind to the cell surface and then transferred to 37°C for 4 hr or 18 hr to detect virus internalization and production of new viral nucleoprotein (NP). To detect NP the cells were washed twice with cold PBS and fixed with 1 ml freshly prepared 3% paraformaldehyde in PBS on ice for 5 min. After washing three times with cold PBS, cells were permeabilized with 2% triton X-100 in PBS for 10 min at room temperature, washed 3 times with cold PBS, once with 50 mM ammonium chloride in PBS to destroy any remaining paraformaldehyde, and blocked with 10% supplemented calf serum in PBS for 15 min at room temperature. 50 µl of rabbit anti-core (mostly anti-NP) antiserum diluted 1∶5000 in 0.1% BSA/PBS was added for 30 min at RT. After washing with 0.1% BSA in PBS, cells were incubated with 50 µl Alexa Fluor-488 labeled goat anti-rabbit antiserum (1∶200 dilution) for 30 min at room temperature. After washing five times with 0.1% BSA/PBS and once with water, the cover slip was drained well and mounted with a small drop of Prolong Antifade reagent with DAPI (Invitrogen-P36931). The slides were analyzed with a Nikon TE-2000E microscope.

**Effect of oseltamivir on virus entry into MDCK cells:** Ten fold dilutions of MDCK-grown virus starting with 10 µl were used to infect MDCK cells in 24 well plates in the absence or presence of 10 µM oseltamivir. The virus +/− inhibitor was adsorbed to cells for 1 hr at room temperature, then unattached virus was aspirated off and 1 ml DMEM-Ham’s F12/ITS+ infection medium containing 0.5 µg/ml TPCK treated trypsin added to each well. After 3 days incubation at 37°C the cytopathic effect (cpe) was estimated by eye and tissue culture infectious titer determined from the last well that had hemagglutinating activity. The same protocol was used with a fixed inoculum of virus (10 µl) and 10-fold dilutions of oseltamivir starting with 10 µM.

## Supporting Information

Figure S1 **Sequence alignment of HA1 of the viruses used in this study.** The alignment was generated by the Influenza Sequence Database [43]. A dot indicates the amino acid is the same as on the top line. Sequons for N-linked glycosylation are highlighted in yellow.
(PDF)

Figure S2 **The isoelectric point (pI) of the HA1 protein of each virus was calculated using EMBOSS Pepstats** [**25**] **and is plotted (green triangles) along with the HAU per µg viral protein (red squares) and glycan array signal (RFU; blue diamonds) per 100** **ng viral protein.**
(PDF)

Table S1 **Glycan array binding data were processed as described in Materials and Methods.** For each glycan, the percentile scores for all viruses were summed, converted to percentile rank, and the 610 glycans sorted from top to bottom score. Aggregate scores less than 15 are considered insignificant and those glycans have been omitted.
(XLSX)
