# Supplementary material for: Human H3N2 Influenza Viruses Isolated from 1968 To 2012 Show Varying Preference for Receptor Substructures with No Apparent Consequences for Disease or Spread
Source: PLoS One. 2013 Jun 21;8(6):e66325. doi: 10.1371/journal.pone.0066325 (PMC3689742; doi:10.1371/journal.pone.0066325)
Supplement: Figure S1 — Sequence alignment of HA1 of the viruses used in this study. The alignment was generated by the Influenza Sequence Database [43]. A dot indicates the amino acid is the same as on the top line. Sequons for N-linked glycosylation are highlighted in yellow. (PDF) [file pone.0066325.s001.pdf]

Figure S1: Sequence alignment of HA1 of the viruses used in this study

```
                             10         20         30         40         50         60         70         80         90        100        110
                              |          |          |          |          |          |          |          |          |          |          |
A/Albany/11/1968     QDLPGNDNST ATLCLGHHAV PNGTLVKTIT NDQIEVTNAT ELVQSSSTGK ICNNPHRILD GIDCTLIDAL LGDPHCDVFQ NETWDLFVER SKAFSNCYPY DVPDYASLRS
A/BCM/2/1968         .......... .......... .......... .......... .......... .......... .......... .......... .......... .......... ..........
A/Albany/1/1969      .......... .......... .......... D......... .......... .......... ..N....... .......... D......... .......... ..........
A/BCM/2/1969         .......... .......... .......... .......... .......... .......... .......... .......... .......... .......... ..........
A/Albany/1/1970      .......... .......... .......... .......... .......... .......... ..N....... .......... D......... .......... ..........
A/BCM/1/1970         .......... .......... .......... .......... .......... .......... ..N....... .......... D......... .......... ..........
A/BCM/1/1972         ..F....... .......... .......... .......... .......... .......... .......... .......G.. .......... .......... ..........
A/BCM/1/1973         ..F....... .......... .......... .......... .......... .......... .......... .......G.. .......... .......... ..........
A/BCM/1/1974         ..F....... .......... ....I..... .......... .......... .......... ..N....... .......G.. .......... .......... Y.........
A/Albany/42/1975     .N........ .......... .......... .......... .......... .......... ..N....... .......G.. ..K....... .......... ..........
A/BCM/3/1975         .......... .......... .......... .......... .......... ..D....... ..N....... .......G.. ..K....... .......... ...G......
A/BCM/1/1976         .......... .......... .......... .......... .......... ..D....... ..N....... .......G.. ..K....... .......... ...G......
A/BCM/11/1976        .N........ .......... .......... .......... .........R ..DS...... .KN....... .......G.. ..K....... .......... ..........
A/BCM/3/1977         .N........ .......... .......... .......... .........R ..DS...... .KN....... .......G.. ..K....... .......... ..........
A/BCM/1/1978         .NF....... .......... .......... .......... .........R ..DS...... .KN....... .......G.. ..K....... .......... ..........
A/BCM/1/1980         .N........ .......... .......... .......... .........R ..DS...... .KN...V... .......G.. ..K....... .......... ..........
A/BCM/1/1981         .N........ .......... .......... .......... .........R ..DS...... .KN...V... .......G.. ..K....... .......... ..........
A/BCM/1/1982         .N........ .......... .......... .......... .........R ..DS...... .KN...V... .......G.. ..K....I.. .......... ..........
A/Memphis/33/83      .K........ .......... .......... .......... .........R ..DS...... .KN....... .......G.. ..K....... .......... ..........
A/Memphis/2/85       .K........ .......... .......... .......... .........R ..DS...... .KN....... .......G.. ..K....... .......... ..........
A/Memphis/2/1986     .K........ .......... .......... .......... .........R ..DS...... .KN....... .......G.. ..K....... .......... ..........
A/Memphis/3/88       .K........ .......... .......... .......... .........R ..DS...... .KN....... .......G.. ..K....... ...Y...... ..........
A/Memphis/7/90       .K........ .......... ....I..... .......... .........R ...S...... .KN....... .......S.. .KE....... ...Y...... ..........
A/BCM/1/1991         .K........ .......... .......... .......... .........R ..DS...... .KN....... .......G.. .KE....... ...Y...... ..........
A/BCM/2/1992         .K........ .......... .......... .......... .........R ..DS...... .KN....... .......G.. .KE....... ...Y...... ..........
A/BCM/1/1993         .K........ .......... .......... .......... .........R ..DS...... .KN....... .......G.. .KE....... ...Y...... ..........
A/Memphis/7/94       .K........ .......... .......... .......... .....F...R ..DS...... .KN....... .......G.. .KE....... ...Y...... ..........
A/New York/696/1994  .K........ .......... .......... .......... ......P..R ..DS...... .KN....... .......G.. .KE....... ...Y...... ..........
A/Memphis/9/95       .K........ .......... .......... .......... ....N.P..R ..DS...... .KN....... .......G.. .KE....... ...Y...... ..........
A/Memphis/9/1996     .K........ .......... .......... .......... .......I.R ..DS...... .KN....... .......G.. .KE....... ...Y...... ..........
A/Oklahoma/3003/96   .K........ .......... .......... .......... ....G....R ..DS...... .KN....... .......G.. .KE....... ...Y...... ..........
A/Oklahoma/5098/96   .K........ .......... .......... .......... .........R ..DS...... .KN....... ..?....G.K .KE....... TT.Y...... ..........
A/Memphis/5/97       .K........ .......... .......... .......... .........R ..DG..Q... .KN....... .......G.. .KE....... ...Y...... ..........
A/Memphis/14/1998    .KI....... .......... .......... .......... .....F...R ..DS...... .EN....... .......G.. .KE....... ...Y...... ..........
A/Memphis/49/99      .K..V..... .......... .......... ..H....... .........R ..DS..Q... .EN....... .......G.. .KE....... .T.Y...... ..........
A/BCM/1/2001         .K........ .......... .......... .......... .........R ..DS..Q... .EN....... .......G.. .KE....... ...Y...... .....V....
A/BCM/1/2002         .K........ .......... .......... .......... ........SR ..DS..Q... .EN....... .......G.. .KE....... ...Y...... .....V....
A/Memphis/27/2003    .K........ .......... ....I..... .......... .........G ..DS..Q... .EN....... ....Q..G.. .KK....... ...Y...... ..........
A/Oklahoma/323/03    .K........ .......... ....I..... .......... .........G ..DS..Q... .EN....... ....Q..G.. .KK....... ...Y...... ..........
A/Oklahoma/1992/05   .K........ .......... ....I..... .......... ..I......G ..DS..Q... .EN....... ....Q..G.. .KK....... ...Y...... ..........
A/Oklahoma/309/06    .K........ .......... ....I..... .......... .........E ..DS..Q... .EN....... ....Q..G.. .KK....... ...Y...... ..........
A/Oklahoma/483/08    .KF....... .......... ....I..... .......... .........E ..DS..Q... .EN....... ....Q..G.. .KN....... ...Y...... ..........
A/Oklahoma/5342/10   .K........ .......... ....I..... .......... .........E ...S..Q... .EN....... ....Q..G.. .KK....... ...H...... ..........
A/Oklahoma/5386/10   .K........ .......... ....I..... .......... .........E ...S..Q... .EN....... ....Q..G.. .KK....... ...H...... ..........
A/Oklahoma/2280/12   .KI....... .......... ....I..... .......... .........E ...S..Q... .EN....... ....Q..G.. .KK....... ...H...... ..........
```

	120	130	140	150	160	170	180	190	200	210	220
A/Albany/11/1968	LVASSGTLEF	ITEGFTWTGV	TQNGGSNACK	RGPGSGFFSR	LNWLTKSGST	YPVLNVTMPN	NDNFDKLYIW	GVHHPSTNQE	QTSLYVQASG	RVTVSTRRSQ	QTIIPNIGSR
A/BCM/2/1968	....	....	....	....	....	....	....	....	....	....	....
A/Albany/1/1969	....	....	....	....	....	....	....	....	....	....	....
A/BCM/2/1969	....	....	....	....	....	....	....	....	....	....	....
A/Albany/1/1970	....	....	....	....	....	....	....	....	....	....	....
A/BCM/1/1970	....	....	....	....	....	....	....	....	....	....	....
A/BCM/1/1972	....	.S........	....	...D......	....Y.....	....	....	.......D..	....	......K...	....
A/BCM/1/1973	....	.N........	....	...D......	....Y.....	....	....	.......D..	....	......K...	....
A/BCM/1/1974	....	.N...N....	....	...N......	....Y...T.	....	....	.......D..	..N.......	......K...	...T......
A/Albany/42/1975	....	.N...N....	....	...D......	....Y.....	....	....	.......D..	..N.......	......K...	......V...
A/BCM/3/1975	....	.N...N....	......S...	...DN.....	....Y.....	...Q......	...S......	.......DK.	..D.......	K.....K...	......V...
A/BCM/1/1976	....	.N...N....	......S...	...DN.....	....Y.....	...Q......	...S......	.......DK.	..D.......	K.....K...	......V...
A/BCM/11/1976	....	.N...N....	......Y...	...DNS....	....YE.E.K	....	....	.......DK.	..K.......	.I....K...	......V...
A/BCM/3/1977	........K.	.N...N....	......Y...	...DNS....	....Y..E..	....	....	.......DK.	..N.......	......K...	......V...
A/BCM/1/1978	....	.N...N....	......Y...	...DNS....	....YE.E.K	....	....	.......DK.	..N.......	......K...	......V...
A/BCM/1/1980	....	.N.S.N....	..S...Y...	..SDNS....	....YE.E.K	....	.G........	.......DK.	..N...R...	......K...	....
A/BCM/1/1981	....	.N.S.N....	..S...Y...	..SDNS....	....YE.E.K	....	.G........	.......DK.	..N...R...	......K...	....
A/BCM/1/1982	....	.N.S.N....	..S...Y...	..SDNS....	....YE.E.K	....	.G........	.......DK.	..N...R...	......K.N.	..V.......
A/Memphis/33/83	....	.N...N....	..S...Y...	..SVNS....	....YE.E.K	....	.GK.......	.......DK.	..N...R...	......K...	..V.......
A/Memphis/2/85	....	.N...N....	..S...Y...	..SVNS....	....YE.E.K	..A.......	.GK.......	.......DK.	..N...R...	......K...	..V.......
A/Memphis/2/1986	....	.N...N....	..S...YT..	..SVNS....	....YE.E.K	..A.......	.GK.......	.......DK.	..N...R...	......K...	..V.....P.
A/Memphis/3/88	....	.N.D.N....	..S...Y...	..SVNS....	....HE.EYK	..A.......	.GK.......	.......DR.	..N...R...	......K...	..V.......
A/Memphis/7/90	....	.N.D.N....	A.S...Y...	..SVNS....	....HE.EYK	..A.......	.GK.......	.......DR.	..N...R...	......K...	..V.......
A/BCM/1/1991	....	TN.D.N....	A.S.E.Y...	..SVKS....	....HE.DYK	..A.......	.GK.......	.......DR.	......R...	......K...	..V.......
A/BCM/2/1992	....	TN.D.N....	A.S...Y...	..SVKS....	....HE.DYK	..A.......	.GK.......	.......DR.	......R...	......K...	..V.......
A/BCM/1/1993	....	.N.D.N....	A.D.K.Y...	..SVNS....	....H.LEYK	..A.......	.GK.......	.......DSD	......R...	......K...	..V.......
A/Memphis/7/94	....	.N.D.N....	A.D.K.Y...	..SVNS....	....H.LEYK	..A.......	.GK.......	.......DSD	......R...	......K...	..V.....F.
A/New York/696/1994	....	TN.N.N....	A.D.K.Y...	..SVNS....	....H.LEYK	..A.......	.GK.......	.......DSD	......R...	K.....K...	..V..D..Y.
A/Memphis/9/95	....	TN.N.N....	A.D.K.Y...	..SVNS....	....H.LEYK	..A.......	.GK.......	.......DSD	......R...	K.....K...	..V..D..Y.
A/Memphis/9/1996	....	TN...N....	A.D.K.Y...	..SVNS....	....H.LEYK	..A.......	..K.......	.......DSD	....	......K...	..V.......
A/Oklahoma/3003/96	....	NN.S.N....	A...T.D...	.RSVKS....	....H.LEYK	..A...A...	..K.......	.......DSV	....	......K...	..V.......
A/Oklahoma/5098/96	.?........	TN...N....	A.D.T.D..R	..SVKS...K	....H.LEYK	..A.K.....	..K.......	.......DSV	....	......K...	..V......?
A/Memphis/5/97	....	TN.S.N....	A...T.Y...	..SVKS....	....H.LEYK	..A.......	..K.......	.......DSD	.......S..	......K...	..VT......
A/Memphis/14/1998	....	NN.S.N....	A...T.Y...	.RSIKS....	....HQLKYK	..A.......	..K.......	.......DSD	.....A....	......K...	..V.......
A/Memphis/49/99	....	NN.S.N....	A...T.S..Q	.RSIKS....	....HQLKYK	..A.......	.EK.......	.......DSD	.I...A....	......K...	..V.......
A/BCM/1/2001	....	NN.S.N....	A...T.S...	.RSDKS....	....HQLKYK	..A.......	.EK.......	.....G.DSD	.I...A....	......K...	..V......G
A/BCM/1/2002	....	NS.S.N....	A...T.S...	.RSDKS....	....HQLKYK	..A.......	.EK.......	.....G.DSD	.I...A....	......K...	..V.......
A/Memphis/27/2003	....	NN.S.D....	....T.S...	.RSNKS....	.....HLKYK	..A.......	.EK.......	.....G.DSD	.I...A....	.I....K...	..V.......
A/Oklahoma/323/03	....	NN.S.D....	....T.S...	.RSNKS....	.....HLKYK	..A.......	.EK.......	.....G.DSD	.I...A....	.I....K...	..V.......
A/Oklahoma/1992/05	....	NN.S.N....	....T.S...	.RSNNS....	.....HLKFK	..A.......	.EK.......	.....G.DND	.I...A....	.I....K...	..V.......
A/Oklahoma/309/06	....	NN.S.N....	....T.SS..	.RSNNS....	.....HLKFK	..A.......	.EK.......	.....G.DND	.IF..A....	.I....K...	..V.......
A/Oklahoma/483/08	....	NN.S.N....	....T.S..I	.RSNNS....	.....H.KFK	..A.......	.E........	.....G.DND	.IF..A....	.I....K...	..V.......
A/Oklahoma/5342/10	....	NN.S.N....	....T.S..I	.RSNNS....	.....HLNFK	..A.......	.EQ.......	.....G.DKD	.IF..A..A.	.I....K...	.AV.......
A/Oklahoma/5386/10	....	NN.S.N....	....T.S..I	.RSNNS....	.....HLNFK	..A.......	.EQ.......	.....G.DKD	.IF..A..A.	.I....K...	.AV.......
A/Oklahoma/2280/12	....	NN.S.N....	....T.S..I	.RSNNS....	.....HLNFK	..A.......	.EQ.......	.....G.DKD	.IF..A..A.	.I....K...	.AV.......

```
                           230        240        250        260        270        280        290        300        310        320        330
                             |          |          |          |          |          |          |          |          |          |          |
A/Albany/11/1968    PWVRGLSSRI SIYWTIVKPG DVLVINSNGN LIAPRGYFKM RTGKSSIMRS DAPIDTCISE CITPNGSIPN DKPFQNVNKI TYGACPKYVK QNTLKLATGM RNVPEKQTRG
A/BCM/2/1968        .......... .......... .......... .......... .......... .......... .......... .......... .......... .......... ..........
A/Albany/1/1969     .......... .......... .......... .......... .......... .......... .......... .......... .......... .......... ..........
A/BCM/2/1969        .......... .......... .......... .......... .......... .......... .......... .......... .......... .......... ..........
A/Albany/1/1970     .......... .......... .......... .......... .......... .......... .......... .......... .......... .......... ..........
A/BCM/1/1970        .......... .......... .......... .......... .......... .......... .......... .......... .......... .......... .........?
A/BCM/1/1972        .......... .......... .I........ .......... .......... ....G..... .......... .......... .......... .......... .........?
A/BCM/1/1973        .......... .......... .I........ .......... .......... ....G..... .......... .......... .......... .......... ..........
A/BCM/1/1974        .......... .......... .I........ .......... .......... ....G..... .......... .......... .......... .......... ..........
A/Albany/42/1975    .......... .......... .I........ ......H... .......... ....G..S.. .......... .......... .......... .......... ..........
A/BCM/3/1975        .........V .......... .I........ .......... .......... ....G..S.. .......... .......... .......... .......... ..........
A/BCM/1/1976        .........V .......... .I........ .......... .......... ....G..S.. .......... .......... .......... .......... ..........
A/BCM/11/1976       .......... .......... .I.L...... .........V .......... ....G..S.. .......... .......... .......... .......... ..........
A/BCM/3/1977        .......... .......... .I.L...... .........I .......... ....G..S.. .......... .......... .......... .......... ........K.
A/BCM/1/1978        .......... .......... .I.L...... .........I .......... ....G..S.. .......... .......... .......... .......... ..........
A/BCM/1/1980        .......... .......... .I.L...... .........I .......... ....G..S.. .......... .......... .......... .......... ..........
A/BCM/1/1981        .......... .......... .I.L...... .........I .......... ....G..S.. .......... .......... .......... .......... ..........
A/BCM/1/1982        .......... .......... .I.L...... .........I .N........ ....G..S.. .......... .......... ...T...... .......... ..........
A/Memphis/33/83     .......... .......... .I.L...T.. .........I .......... ....G..S.. .......... .......... ......R... .......... ..........
A/Memphis/2/85      .......... .......... .I.L...T.. .........I .......... ....G..S.. .......... .......... ......R... .......... ..........
A/Memphis/2/1986    .......... .......... .I.L...T.. .........I .......... ....G..S.G .......... .......... ......R... .......... ..........
A/Memphis/3/88      .......... .......... .I.L...T.. .........I .......... ....G..S.. .......... .......... ......R... .......... ..........
A/Memphis/7/90      .......... .......... .I.L...T.. .........I .......... ....G..S.. .......... ........R. ......R... .......... ..........
A/BCM/1/1991        .......... .......... .I.L...T.. .........I .......... ....G..S.. .......... ........R. ......R... .......... ..........
A/BCM/2/1992        .......... .......... .I.L...T.. .........I .......... ....G..S.. .......... ........R. ......R... .......... ..........
A/BCM/1/1993        .......... .......... .I.L...T.. .........I .N........ ....GN.R.. .......... .......... ......R..R .......... ..........
A/Memphis/7/94      .......... .......... .I.L...T.. .........I .N........ ....GN.S.. .......... ........R. ......R... .......... ..........
A/New York/696/1994 .......... .......... .I.L...T.. .........I .N........ ....GN.S.. .......... ........R. ......R... .......... ..........
A/Memphis/9/95      .......... .......... .I.L...T.. .........I .N........ ....GN.S.. .......... ........R. ......R... .......... ..........
A/Memphis/9/1996    .....I.... .......... .I.L...T.. .........I .N........ .....N.N.. .......... ........R. ......R... .......... ..........
A/Oklahoma/3003/96  .....I.... .......... .I.L...T.. .........I .S........ .....N.N.. .......... ........R. ......R... .......... ..........
A/Oklahoma/5098/96  .....V.... .......... .I.L...T.. .........I .S...V.... .....N.N.. .......... ........R. ......R..R .......... ..........
A/Memphis/5/97      .....V.... .......... .I.L...T.. .........I .R........ .....N.N.. .......... ........R. ......R... .......... ..........
A/Memphis/14/1998   .....V.... .......... .I.L...T.. .........I .S........ ....GK.N.. .......... ........R. ......R... .......... ..........
A/Memphis/49/99     .....V.... .......... .I.L...T.. .........I .S........ N...GK.N.. .......... ........R. ......R... .......... ..........
A/BCM/1/2001        .....V.... .......... .I.L...T.. .........I .S........ ....GK.N.. .......... ........R. ...P..R... .......... ..........
A/BCM/1/2002        ..G..V.... .......... .I.L...T.. .........I .S........ ....GK.N.. .......... ........R. ......R... .......... ..........
A/Memphis/27/2003   .R..DV.... .......... .I.L...T.. .........I .S........ ....GK.N.. .......... ........R. ......R.I. .......... ..........
A/Oklahoma/323/03   .R..DI.... .......... .I.L...T.. .........I .S........ ....GK.N.. .......... ........R. ......R... .......... ..........
A/Oklahoma/1992/05  .R..DIP... .......... .I.L...T.. .........I .S........ ....GK.N.. .......... ........R. ......R... .......... ..........
A/Oklahoma/309/06   .R..NIP... .......... .I.L...T.. .........I .S........ ....GK.N.. .......... ........R. ......R... .......... ..........
A/Oklahoma/483/08   .R..DIP... .......... .I.L...T.. .........I .S........ ....GK.N.. .......... ........R. ......R... .......... ..........
A/Oklahoma/5342/10  .R..DIP..V .......... .I.L...T.. .........I .S........ ....GK.N.A .......... ........R. ......R... .......... ..........
A/Oklahoma/5386/10  .R..DIP..V .......... .I.L...T.. .........I .S........ ....GK.N.A .......... ........R. ......R... .......... ..........
A/Oklahoma/2280/12  .R..NIP..V .......... .I.L...T.. .........I .S........ ....GK.N.A .......... ........R. ......R... .......... ..........
```

Generated by the Influenza Sequence Database

Macken, C., Lu, H., Goodman, J., & Boykin, L., "The value of a database in surveillance and vaccine selection." in *Options for the Control of Influenza IV.* A.D.M.E. Osterhaus, N. Cox & A.W. Hampson (Eds.) Amsterdam: Elsevier Science, 2001, 103-106.

A dot indicates the amino acid is the same as on the top line. Sequons for N-linked glycosylation are highlighted in yellow.
