# Supplementary material for: Human H3N2 Influenza Viruses Isolated from 1968 To 2012 Show Varying Preference for Receptor Substructures with No Apparent Consequences for Disease or Spread
Source: PLoS One. 2013 Jun 21;8(6):e66325. doi: 10.1371/journal.pone.0066325 (PMC3689742; doi:10.1371/journal.pone.0066325)
Supplement: Figure S2 — The isoelectric point (pI) of the HA1 protein of each virus was calculated using EMBOSS Pepstats [25] and is plotted (green triangles) along with the HAU per µg viral protein (red squares) and glycan array signal (RFU; blue diamonds) per 100 ng viral protein. (PDF) [file pone.0066325.s002.pdf]

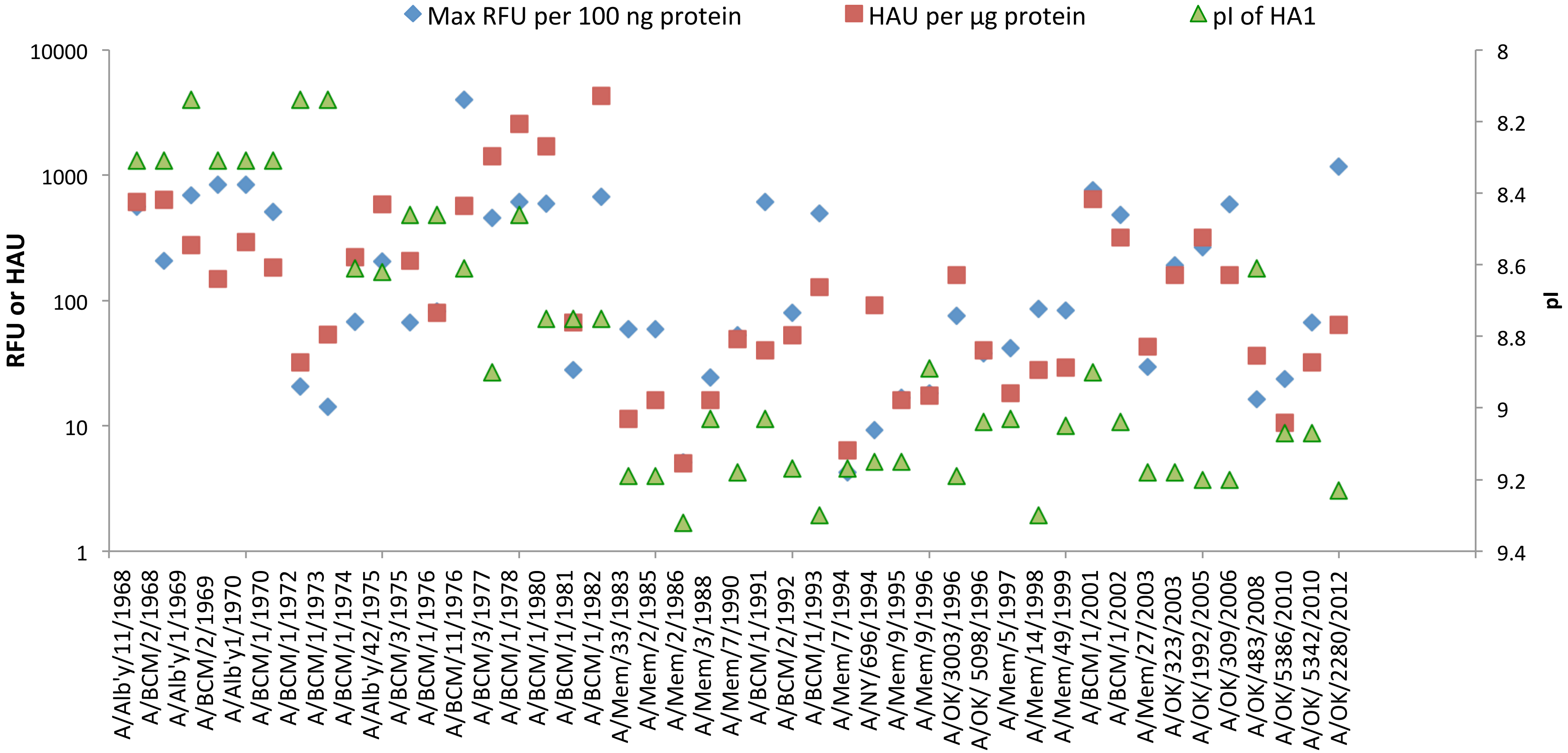

Supplementary Figure 2: The isoelectric point (pI) of the HA1 protein of each virus was calculated using EMBOSS Pepstats and plotted (green triangles) along with the HAU per µg viral protein (red squares) and glycan array signal (RFU; blue diamonds) per 100 ng viral protein.
